# Evaluation of Adolescents’ Awareness of Seat Belt Use and the Relationship with Risky Behaviors

**DOI:** 10.3390/children11060656

**Published:** 2024-05-28

**Authors:** Hatice Topal, Sadettin Burak Açıkel, Hülya Şirin, Emine Polat, Harun Terin, Mehmet Mustafa Yılmaz, Saliha Şenel

**Affiliations:** 1 Department of Pediatrics, Faculty of Medicine, Muğla Sıtkı Koçman University, 48000 Muğla, Türkiye; haticetopal@mu.edu.tr; 2 Department of Child and Adolescent Psychiatry, School of Medicine, Ankara University, 06260 Ankara, Türkiye; 3 Department of Public Health, Faculty of Medicine, University of Health Sciences Turkey, 06010 Ankara, Türkiye; hulya.sirin@sbu.edu.tr; 4 Department of Pediatrics, Dr. Sami Ulus Training and Research Hospital, 06010 Ankara, Türkiye; emine.polat@sbu.edu.tr (E.P.); harun.terin@saglik.gov.tr (H.T.); mustafayilmaz@ankara.edu.tr (M.M.Y.); salihasenel@aybu.edu.tr (S.Ş.); 5 Department of Pediatrics, Faculty of Medicine, Ankara Yıldırım Beyazıt University, 06800 Ankara, Türkiye

**Keywords:** traffic, accident, risk, behavior, adolescent

## Abstract

The one of the leading causes of adolescent morbidity and mortality worldwide is motor vehicle accidents (MVA). The use of seat belts significantly lowers MVA fatalities and injuries. The aim of this study is to investigate adolescent seat belt usage patterns and relation with risky behaviors. The study conducted at two medical institutions with non-immigrant and literate adolescents aged 12–18. Demographics, seat belt use, and risk-taking behavior were collected through questionnaires. 726 teenagers (422 girls and 304 boys) with an average age of 176.7 ± 23.37 months participated in the study. Parents’ educational levels and front-seat belt use have been found to be correlated. Comparatively to non-users, seat belt users demonstrated lower risk scores (total, traffic, substance, and social). The use of seat belts was significantly predicted by traffic risk, according to logistic regression. The frequency of seatbelt use was higher among participants from cities with higher socioeconomic status. As a result, it was found that adolescents who exhibited more risky behaviors had a lower frequency of seat belt use and seat belt use was associated with socioeconomic level and parental education level. It is thought that population-based studies to be conducted on this subject are important.

## 1. Introduction

Motor vehicle accidents (MVA) are the most common cause of morbidity and mortality worldwide in adolescents either riding as a passenger or a driver [1] The use of seat belts is the single most effective way of reducing serious injury and death in MVA [2]. Increasing the seat belt rate by 1% will reduce the number of deaths per 100 thousand vehicles by 1.2 people [3]. There is strong evidence that enforcement programs for safety belt laws increase seat belt use and reduce traffic fatalities in the general population [4]. According to the Highway Traffic Regulation valid in Turkey, it is obligatory to have and wear seat belts and to have a child restraint system [5].

Sociocultural level and regional differences in seat belt use are not well described. However, observational seat belt studies and fatal crash data indicated that seat belt use rates in rural areas were slightly lower than urban areas in all ages [6].

Adolescence, the period between childhood and emerging adulthood, is characterized by increased sensation seeking and risk-taking behavior such as alcohol and drug use, cigarette smoking, and unprotected sex [7,8]. Risky driving behaviors among teens, including non use of seat belts, are known to co-occur with these risky behaviors. However, the most common risky behaviors that co-occur with seat belt nonuse among the adolescents are not known exactly because of the non-representative samples of previous studies [9,10]. Motor vehicle accidents are also the leading cause of death in the adolescent age group in our country according to Turkish Statistical Institute data.

Based on this data, this study was planned to evaluate the habits of adolescents about seat belt use and to determine its relationship with risky behaviors in two centers with different sociocultural and environmental characteristics by using Adolescent Risk-Taking Questionnaire. The main goal of the study was to investigate the possible association between adolescents’ risk-taking behaviors and their seat belt use habits. It was also aimed to investigate different sub-dimensions of risk-taking behavior and seat belt usage habits. Other goals of the study are to evaluate the association between seat belt use and variables such as age and gender, and to determine the situation in cities with different socioeconomic development levels. To our knowledge there is no study that investigates the use of seat belts and risk-taking behaviors of adolescents with quantitative measurements. This study will contribute valuable data to the limited number of literature and can help as a guide in terms of precautions to be taken in this regard.

## 2. Materials and Methods

The research is a prospective, cross-sectional, observational, non-control group study. The study was conducted at Ankara Dr Sami Ulus Maternity, Children’s Health and Diseases Training and Research Hospital and Muğla Sıtkı Koçman University Faculty of Medicine. The study included Turkish adolescents between the ages of 12–18 who applied to the general pediatric outpatient clinic and volunteered to participate in the study. Adolescents who were immigrants in our country, were illiterate, had neuro-metabolic, developmental retardation, psychiatric and chronic diseases, had hearing and vision problems, and could not obtain consent were excluded from the study. All participants who matched the inclusion criteria were included in the study consecutively. There were no missing data and none of the participants were excluded. This study was performed in line with the principles of the Declaration of Helsinki. Approval was granted by the Ethics Committee of Dr Sami Ulus Maternity Children’s Health and Diseases Training and Research Hospital (ID: 2021/03-129). Written informed consent was obtained from the participants and legal guardians. and questionnaire forms were given. The demographic characteristics and seat belt usage habits of the patients were collected with the sociodemographic data form created by the researchers. In addition, Adolescent Risk-Taking Scale was applied in adolescents.

Sociodemographic Data Form: This form has been designed by the researchers for use in this study. In this form, participants were asked about their age, gender, educational status, age, education level and occupation of mother and father. In addition, questions were asked about seat belt usage habits. It was asked whether a seat belt was used while sitting in the front seat and in the back seat of motor vehicles. Participants were asked to answer these questions over 5 options (5 = Always, often, sometimes, rarely, 1 = never). This data evaluated as an ordinal data and used in correlation analysis. Among participants, those who answer, “Do you wear a seat belt when traveling in the front seat?” and “Do you wear a seat belt when traveling in the back seat?” as sometimes, rarely, never are categorized as “seatbelt non-users”, and those who answer always and often as “seatbelt users”.

Adolescent Risk-Taking Questionnaire (ARTS): Adolescent Risk-Taking Questionnaire is a scale developed to measure the risk-taking behavior of students in adolescence. This scale was developed by Gullone et al. [11]. and adapted Turkish by Kıran et al. [12]. The items of the Adolescent Risk Taking Scale are a 5-point Likert-type scale that shows various behaviors that adolescents take risks. Individuals are asked to choose one of the options “I always do”, “I do it often”, “I do it sometimes”, “I do it very little”, “I do not do it at all”. The highest score that can be obtained from the scale is 170 and the lowest score is 34. The increase in the score obtained from the scale indicates that the risk-taking behavior on students is high. There are three subscales of Turkish version (social risk, traffic risk, substance use risk.) The internal consistency coefficient for all 26 items of the scale is 0.88. The alpha coefficient was 0.84 for the risk-taking subscale related to social position, 0.74 for the traffic-related risk-taking subscale consisting of 6 items, and 0.62 for the risk-taking subscale consisting of 5 items. The reliability coefficient obtained as a result of the test-retest study was found to be 0.85 for the whole scale, 0.76 for social risk, 0.67 for the traffic risk, and 0.64 for the substance use risk subscale.

SPSS 26.0 software was used to perform statistical analysis on the collected data. Demographic variables were analyzed using descriptive statistics. The Kolmogorov-Smirnov test was used to determine the data’s normality. To examine the differences between the groups, the independent sample *t* test was used or Mann-Whitney U test were used. To examine the differences between categorical variables, the Chi-square test was used. The spearman correlation analysis was used to correlation and logistic regression was used for determining the predictors of seat belt use. Levels of significance was determined as 0.05.

## 3. Results

The study sample was consisted of 726 adolescents (422 females, 304 males). The mean age of sample was 176.7 ± 23.37 months. There was no statistically significant difference between females and males in terms of age (*p* = 0.358, t = 0.919). There was no statistically significant difference between females and males in terms of total (*p* = 0.182) traffic risk (*p* = 0.212), substance abuse risk (*p* = 0.11) and social risk (*p* = 0.237) scores. Detailed statistics were given in Table 1.

There was statistically significant positive correlation between mother’s educational level and using seat belt in front seat (r = 0.184, *p* < 0.001) and father’s educational level (r = 0.208, *p* < 0.001). There was was statistically significant negative correlation between age and using seat belt in back seat (r = −0.083, *p* = 0.026).

When the two study centers were compared in terms of seat belt use, it was observed that the frequency of seat belt use in the front seat was significantly higher in Muğla. No difference was observed in seat belt use in the back seat. Detailed statistics were given in Table 2.

There was statistically significant difference between seat belt users and seat belt non- users in terms of total (U = 36,459.5, *p* < 0.001), traffic risk (U = 34,117.5, *p* < 0.001), substance abuse risk (U = 42,754, *p* < 0.001) and social risk (U = 38,590, *p* < 0.001) scores for front seat. There was also statistically significant difference between seat belt users and seat belt non users in terms of total (U = 40,713.5, *p* < 0.001), traffic risk (U = 38,069, *p* < 0.001), substance abuse risk (U = 51,162, *p* < 0.001) and social risk (U = 42,823.5, *p* < 0.001) scores for back seat. Significant negative correlation was found between total, traffic, substance abuse, social risk scores and the frequency of seat belt use in front seat and back seat. Detailed correlation statistics were given in Table 3.

Predictors of using seat belt in front and back seat were assessed with logistic regression analysis. Using seat belt was defined as the dependent variable (0 = non-user 1 = users) and gender, age, social, traffic, substance abuse risk scores were defined as independent variables. According to this analysis, traffic risk scores was a significant predictor of using seatbelt in front seat (Cox-Snell R^2^ = 0.057, *p* < 0.001, odds ratio 0.774), and back seat (Cox-Snell R^2^ = 0.063, *p* < 0.001, odds ratio: 0.732). The results of logistic regression analysis are shown in Table 4.

## 4. Discussion

In this study, we have investigated the association between adolescent seatbelt use and risky behavior in a large sample. According to results, increased risk scores were detected among seatbelt nonusers and traffic risk scores was found a significant predictor of seatbelt use. Our results were consistent with the literature and repeated this data in a different way.

Malekpour et al. reported in their study that, the rate of seat belt use in the front seat inside the city was lower than that outside the city. These researchers designed their studies within the framework of Theory of Planned Behavior (TPB). According to results, in terms of TPB constructs, perceived behavioral control, subjective norm, and attitude all had a significant and positive relationship with the intention to use a seat belt. Furthermore, behavioral intention was found to have a significant positive relationship with seat belt use [13]. We have also found that traffic risk scores were a significant predictor of seatbelt use in both front seat and back seat. Our study is differentiated mentioned study with larger sample size and evaluating both front seat and back seat. Jiang et al. also conducted a study within the framework of Theory of Planned Behavior (TPB) among participants between 16–25 age. The findings demonstrated that only the subjective norms had no statistically significant difference between males and females among the six psychological factors that influence seat belt usage behavior in the front seat. Additionally, there are notable differences between males and females in the six psychological factors that influence seat belt usage behavior in the back seat. The findings also revealed that, in addition to perceived behavioral control, there were significant differences between transportation majors and students with other majors in the scores of the standard TPB variables, the extended variables, and behavioral intention. The transportation majors tended to use seat belts more frequently and had a more positive attitude toward the value of seat belts in reducing the severity of accidents [14]. In our study, we found that seat belt use decreased as risk-taking tendency increased. From this point of view, the results of the mentioned study are consistent with the results of our study.

To learn more about the correlates of seat belt use, a cross-sectional study of 1077 students enrolled in a large, comprehensive Midwestern university in the United States was conducted about this subject. Seven risk-taking behaviors, health-promoting behaviors, age, sex, and race were all examined. Overall, there was an inverse relationship between the frequency of seat belt use and five of the seven risky behaviors, including the use of drugs, drinking while driving, and smoking cigarettes. Seat belt use was positively associated with health-promoting behavior, as determined by a 48-item index [15]. In a similar study, lack of seat belt use was linked to increased alcohol, cigarette, marijuana, and cocaine use, increased tolerance for speeding and drinking while driving, decreased exercise, and increased preference for dietary fat [16].

The relationship between demographic parameters such as age and education level and safety use has been previously investigated in the literature. In a recent study, the percentage of adolescents who reported not wearing seat belts increased with age [17]. In another study, when other covariates were taken into account, those between the ages of 26 and 45 were 49% more likely to use a seatbelt than those between the ages of 18 and 25 [18]. Also, it was mentioned that more educated individuals are more likely to use seatbelts and understand their importance [19]. Consistent with all these data, we found that during adolescence, when risk-taking behavior increases, the frequency of seat belt use in the back seat decreases as the age of the adolescent increases. The reason why we found this relationship only in the back seat may be that seat belt use in the back seat is more associated with adherence to rules. In addition, we found that the frequency of seat belt use in both front and back seats increased as parental education level increased. Our study is important in terms of investigating the relationship between parental education level and adolescent seat belt use.

When the two study centers were compared in terms of seat belt use, it was observed that the frequency of seat belt use in the front seat was significantly higher in Muğla. No difference was observed in seat belt use in the back seat. We think that this difference may be due to the fact that Muğla (26th) is ahead of Ankara (44th) in the field of education in the Rankings and index values of well-being index for provinces survey conducted by the Turkish Statistical Institute [20]. In a study about this association, it was found that, seat belt usage increased as the education level increases [21].

This study has certain limitations. Cross-sectional data collection with scales and sampling from hospitals may not reflect the population and the results cannot generalized to the population. We did not collect any data about parents’ driving practices. If we did, we could include this data during the analysis. Nevertheless, interesting findings adding knowledge to the scanty literature about seat belt use with large sample size; measuring risky behaviors with a structured scale and investigating the relationship between demographic data which have not been studied before are the major strengths of this study.

## 5. Conclusions

Parental educational status positively correlated with seat belt use in the front seat whereas seat belt use in the back seat decreased by age. All risk scores including total risk score, traffic risk score, substance abuse risk score and social risk score were significantly lower in seat belt users. This may be related to the fact that parents with higher educational levels have positive approach to encourage seat belt usage and to prevent risky behaviors in children. The finding of higher seat belt use in the front seat in Muğla where it is a city with higher sociocultural level may confirm this comment. The seat belt use in the back seat seems not to be accepted and preferred generally regardless of sociocultural and educational level. Parental education about ‘seat belt use’ in our population may positively effect adolescents’ behavior.

If the practical implications of the study are evaluated, since there is a possible correlation between parental education level and seat belt use, remedial interventions to be made regarding the education level of individuals may be beneficial in reducing seat belt use and indirectly reducing the morbidity and mortality rates related to traffic accidents. Adolescence is a period in which risky behaviors increase. According to results, it is thought that traffic-related risky behaviors could determine seat belt use. For this reason, it may be useful to conduct a risk screening before issuing a driver’s license and to provide additional training to individuals with high-risk behaviors.

## Figures and Tables

**Table 1:** Demographic Characteristics of Sample and Comparasion of Adolescent Risk-Taking Questionnaire Subscale Scores Between Genders.

	**Male**		**Female**		**Statistics**	
	**Mean**	**SD**	**Mean**	**SD**	**t**	**p**
Age	175.73	23.98	177.35	22.91	0.919	0.358
	**Mean Rank**	**Sum of Rank**	**Mean Rank**	**Sum of Rank**	**U**	**p**
Total Risk Score	375.69	114,210.50	354.72	149,690.50	60,437.5	0.182
Public Risk Score	374.30	113,788	355.72	150,113	60,860	0.237
Traffic Risk Score	374.39	113,815	355.65	150,086	60,833	0.212
Substance Abuse Risk Score	373.71	113,606.5	356.15	150,294.5	61,041.5	0.11
	**Father**		**Mother**			
	**Mean**	**SD**	**Mean**	**SD**		
Age (Years)	45.49	6.01	42.09	5.91		
Educational Level						
Illiterate	17 (2.3%)		28 (3.9%)			
Primary School Graduated	243 (33.5%)		290 (39.9%)			
High School Graduated	209 (28.8%)		178 (24.5%)			
University Graduated	207 (28.5%)		194 (26.7%)			
Postgraduate	50 (6.9%)		36 (5%)			

**Table 2:** Comparasion of seatbelt use frequency between study centers.

	Ankara		Muğla			
	Yes	No	Yes	No	X^2^	*p*
Using seat belt on front seat	230 (66.9%)	114 (33.1%)	314 (82.2%)	68 (17.8%)	22.670	<0.001
Using seat belt on back seat	110 (32%)	234 (68%)	128 (33.5%)	254 (66.5%)	0.193	0.661

**Table 3:** The correlation between Adolescent Risk-Taking Questionnaire Subscale Scores and frequency of seat belt use.

	Front Seat	Back Seat
Total Risk Score	−0.219 **	−0.285 **
Public Risk Score	−0.189 **	−0.256 **
Traffic Risk Score	−0.265 **	−0.312 **
Substance Abuse Risk Score	−0.163 **	−0.202 **

** Correlation is significant at the 0.01 level.

**Table 4:** Logistic Regression Analysis about predictor of seatbelt use.

**(a). Logistic Regression Analysis about predictor of seat belt use in front seat**							
	**β**	**SE β**	**Wald’s χ^2^**	**p**	**OR**	**95% CI OR** **Lower-Upper**	
Public Risk Score	−0.007	0.016	0.193	0.661	0.993	0.963	1.024
Traffic Risk Score	−0.256	0.064	16.025	<0.001	0.774	0.683	0.878
Substance Abuse Risk Score	−0.012	0.083	0.022	0.882	0.988	0.840	1.162
Gender	0.269	0.183	2.162	0.141	0.764	0.533	1.094
Age	−0.004	0.004	0.889	0.346	0.996	0.989	1.004
Constant	2.353	0.713	10.889	0.001	10.520		
The dependent variable in this analysis is using seatbelt in front seat so that 0 = non-user and 1 = user, gender: 0 = female, 1 = male OR = odds ratio. CI = confidence interval							
**(b). Logistic Regression Analysis about predictor of seat belt use in back seat**							
	**β**	**SE β**	**Wald’s χ^2^**	**p**	**OR**	**95% CI OR** **Lower-Upper**	
Public Risk Score	−0.014	0.017	0.691	0.406	0.986	0.955	1.019
Traffic Risk Score	−0.312	0.077	16.494	<0.001	0.732	0.630	0.851
Substance Abuse Risk Score	−0.040	0.098	0.167	0.683	0.961	0.793	1.164
Gender	−0.265	0.165	2.559	0.110	0.768	0.555	1.061
Age	−0.007	0.004	3.343	0.067	0.993	0.986	1.000
Constant	1.052	0.644	2.668	0.450	2.862		
The dependent variable in this analysis is using seatbelt in back seat so that 0 = non-user and 1 = user, gender: 0 = female, 1 = male OR = odds ratio. CI = confidence interval							

## Data Availability

Data sharing not applicable. The data are not publicly available due to ethical restrictions.
